# Cross-Modality Deep Learning Denoising for Low-Dose μSPECT: Transfer of PET-Trained U‑Net and Diffusion Models

**DOI:** 10.1021/cbmi.5c00270

**Published:** 2026-03-26

**Authors:** Elise Taragola, Sara Neyt, Boxiao Yu, Maya Abi Akl, Boris Vervenne, Kuang Gong, Stefaan Vandenberghe, Christian Vanhove, Florence M. Muller

**Affiliations:** † Medical Image and Signal Processing, Department of Electronics and Information Systems, Faculty of Engineering and Architecture, 86795 Ghent University, Ghent 9000, Belgium; ‡ J. Crayton Pruitt Family Department of Biomedical Engineering, 3463 University of Florida, Gainesville, Florida 32611, United States; § Department of Radiology, Perelman School of Medicine, University of Pennsylvania, Philadelphia, Pennsylvania 19104, United States

**Keywords:** deep learning, denoising diffusion probabilistic models, dose reduction, image denoising, micro-SPECT, transfer-learning

## Abstract

Reducing radiation dose in microsingle photon emission computed tomography (μSPECT) is essential to limit adverse biological effects in small animal studies, especially for longitudinal experiments. However, dose reduction increases noise and degrades image quality. While deep learning-based denoising (DL-DN) has shown promise for low-dose imaging, most prior work has focused on positron emission tomography (PET). DL-DN for μSPECT remains largely unexplored. In this study, we evaluate postreconstruction DL-DN for low-count μSPECT using two 2D architectures: a U-Net and a denoising diffusion probabilistic model (DDPM). To mitigate the limited availability of preclinical training data, we investigated transfer-learning from PET, a related molecular imaging modality, using models pretrained on both μPET (mouse) and clinical PET (human) data. These models were evaluated in a zero-shot setting and further adapted to the μSPECT domain via transfer-learning. Their performance was compared to models trained from scratch using varying amounts of μSPECT data (4–32 volumes). The data set consisted of in vivo mouse scans acquired with two tracers at 10%, 25%, and 50% of standard counts. Both architectures achieved meaningful noise reduction and improved quantitative agreement with standard-count references. Zero-shot PET-pretrained models demonstrated cross-modality generalization, reducing root mean squared error (RMSE) by more than 25% at the lowest count level, although residual artifacts remained due to differences between the PET source and μSPECT target domains. Transfer-learning consistently improved performance, yielding additional RMSE reductions exceeding 5% relative to zero-shot models. Its advantage over scratch-training decreased with increasing data set size, with maximal RMSE gains dropping from over 10% for small training sets to below 3% for larger data sets. Overall, the U-Net provided more robust performance with lower computational cost. These results indicate that DL-DN can support dose reduction in μSPECT, and that transfer-learning from PET offers a practical approach to address the small data set sizes common for preclinical settings.

## Graphical abstract

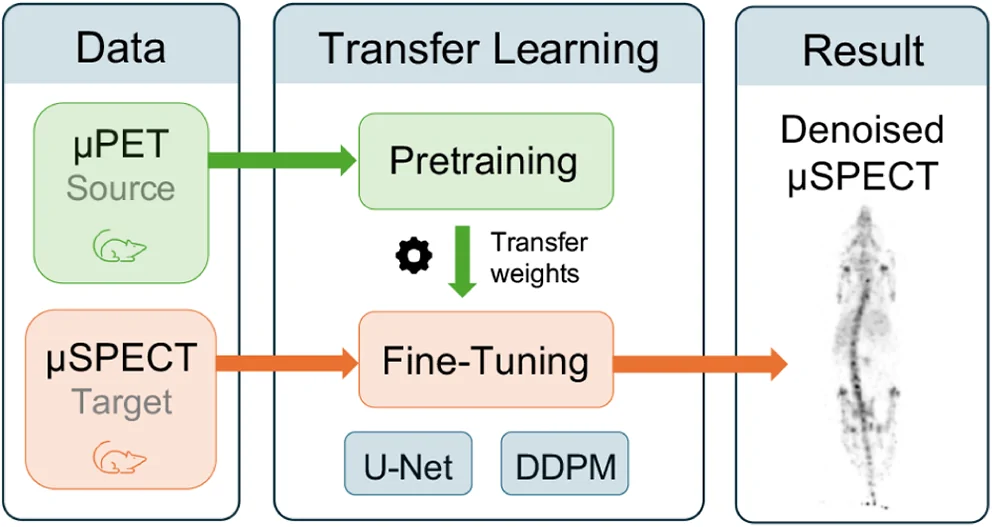

## Introduction

Single photon emission computed tomography (SPECT) is a functional imaging technique that enables noninvasive imaging of molecular processes using radiopharmaceutical tracers. Micro-SPECT (μSPECT) is a miniaturized form of SPECT optimized for small animal imaging with improved sensitivity and resolution compared to clinical systems. It is a valuable tool in translational research of oncology, cardiology, neurology, and drug development, among others.^1^ Recently, μSPECT has also gained increased attention for its role in theranostic approaches, where diagnostic imaging is combined with targeted therapies to personalize treatment and monitor therapeutic responses.^2^ Preclinical imaging in these application fields has multiple advantages. It enables longitudinal monitoring of disease progression and therapeutic response under in vivo conditions. Since each animal can serve as its own control, biological variability is reduced, enhancing study reliability.^3^ This also reduces and refines the usage of animals in preclinical research, supporting the 3R principle (Replacement, Reduction, Refinement).^4^

High image quality is important in preclinical investigations because it enables clear interpretation of tracer distribution patterns and facilitates consistent quantitative evaluation of uptake in specific regions. In μSPECT, high image quality can be achieved through the use of dedicated collimators that provide improved spatial resolution and high sensitivity. Due to the small size of preclinical subjects, substantially higher spatial resolution is required to obtain similar discriminatory capabilities as in humans.^5^ However, achieving submillimeter resolution typically comes at the cost of reduced sensitivity. Higher tracer activities are required to preserve an adequate signal-to-noise ratio (SNR). While administered doses usually remain below lethal thresholds (delivered doses range from 60 to 1000 mGy,^6^ with lethal thresholds around 4.5 Gy for mice^7^), their effect may still affect biological pathways. These risks are particularly relevant in longitudinal studies, where animals are scanned at multiple time points, such as those used for dosimetry in theranostic applications. Additionally, μSPECT is often combined with microcomputed tomography (μCT) to enable anatomical colocalization of tracer uptake and attenuation correction, contributing further to the cumulative dose.^8^ This elevated exposure may induce adverse biological effects and interfere with the physiological processes under investigation.^9^

In line with the ALARA (As Low As Reasonably Achievable) principle,^10^ minimizing radiation dose remains essential for both ethical reasons and research integrity. However, reducing the administered activity in μSPECT lowers the number of emitted and subsequently detected photons. This decline in photon statistics leads to a reduced SNR, which degrades image quality and limits the interpretation of the scans. The increased noise also complicates downstream postprocessing tasks such as segmentation. Additionally, for many radiotracers, only a small fraction is taken up by target tissues, while the majority is excreted via either the liver or the kidneys. This limited specific uptake further reduces the number of detected photons in regions of interest, amplifying the challenges posed by low-dose imaging. While longer acquisition times may compensate for reduced count statistics, they increase the likelihood of animal motion during the scan.

A variety of denoising methods have been developed to counteract these limitations.^11^ Most methods work in the image domain, after image reconstruction as a postprocessing step, as this does not require access to projection data and is therefore compatible with routine imaging workflows. Conventional approaches typically rely on filtering, in which the reconstructed images are convolved with predefined kernels. Although effective in reducing noise, such filter-based methods often blur small structures. As a result, the primary challenge remains to suppress noise while preserving fine functional features that are critical for accurate image interpretation and segmentation. Recent developments in deep learning (DL) have introduced new methods for denoising that overcome some of these limitations.

Although DL-based denoising has been explored for clinical SPECT,^12^ research remains limited compared to other imaging modalities such as CT^13^ and positron emission tomography (PET).^12^ The relative research contributions for the different subfields of medical imaging are shown in Figure 1a. In clinical PET denoising, a broad spectrum of DL architectures has been investigated. Early work focused on relatively simple convolutional designs such as CNNs, convolutional autoencoders, and ResNet variants. This was followed by the widespread adoption of U-Net architectures, which remain a strong and consistently performing baseline. More complex approaches such as generative adversarial networks (GANs) and transformer-based models have also been introduced.^14,15^ Recently, diffusion models, particularly denoising diffusion probabilistic models (DDPMs), have gained increasing attention as a promising generative framework for PET denoising.^16,17^ Clinical SPECT denoising has followed a similar architectural trajectory as PET, yet at a slower pace,^18,19^ with first demonstrations of diffusion models appearing only recently.^20^

**Figure 1:**
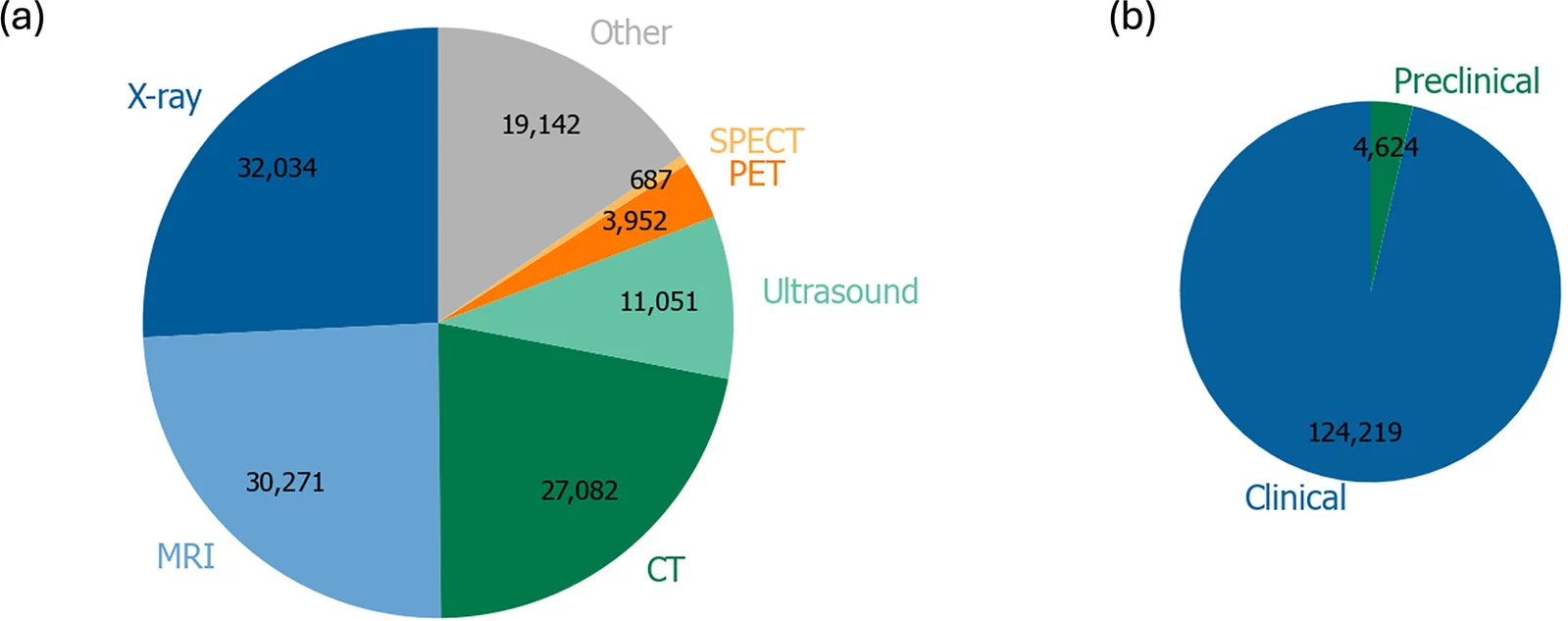
(a) Distribution of publications involving DL across major medical imaging modalities. (b) Distribution of publications in preclinical and clinical domain. Counts were derived from Scopus using queries that search for selected terms within the title, abstract, and keywords, combining DL and modality-specific or domain-specific terminology.

In the literature, U-Net and DDPM-based models represent the most common and emerging approaches in DL-based denoising. U-Nets use an encoder-decoder structure with skip connections to integrate multiscale features, yielding strong performance for many medical imaging tasks.^21^ In contrast, DDPMs follow a generative diffusion process in which images are iteratively refined through a sequence of learned denoising steps rather than a single forward pass. Although DDPMs often employ a U-Net-like backbone for the denoising steps, the diffusion framework allows them to model more complex noise distributions.^22,23^ Recent PET studies report that DDPMs can outperform U-Nets in suppressing low-count noise,^24–26^ although they typically require longer training and inference times.^27^

DL research in preclinical imaging, especially μSPECT, remains underrepresented in literature as shown in Figure 1b. A limited number of studies in μPET have applied U-Net-based architectures^28^ or attention-enhanced CNN variants,^29^ but the methodological diversity remains limited. To our knowledge, no DL-based denoising approaches have been reported for μSPECT, even though this preclinical imaging modality remains a key tool in translational research and plays a growing role in theranostic applications. A major barrier is the small size of typical preclinical data sets, which hampers the data-hungry training of deep networks. Transfer-learning provides a potential route to mitigate this constraint by allowing models to reuse feature representations from larger or better-established data sets.^30^ PET-trained networks represent a natural source domain, as PET and SPECT are both molecular imaging modalities based on the detection of photons emitted by radioisotopes from within the body of the test subject. At the same time, the modalities differ in photon energy, tracer distribution, detector design, and collimation, which influence sensitivity, spatial resolution, and the overall appearance of reconstructed images.^31^ These differences motivate evaluating whether PET-pretrained denoising networks can be effectively adapted to μSPECT.

In this study, we investigate postreconstruction DL-based denoising for μSPECT. Specifically, we evaluate U-Net and DDPM models as representative architectures. We compare models trained from scratch on μSPECT data with models adapted via transfer-learning from μPET-pretrained networks, and additionally evaluate their zero-shot performance on μSPECT data. Figure 2 provides a visual summary of the different training strategies. To further explore the impact of source domain alignment, we also investigate the feasibility of cross-species transfer-learning using networks pretrained on clinical (human) PET data. In addition, we investigate how the number of μSPECT training data sets affects the relative performance of these strategies.

**Figure 2:**
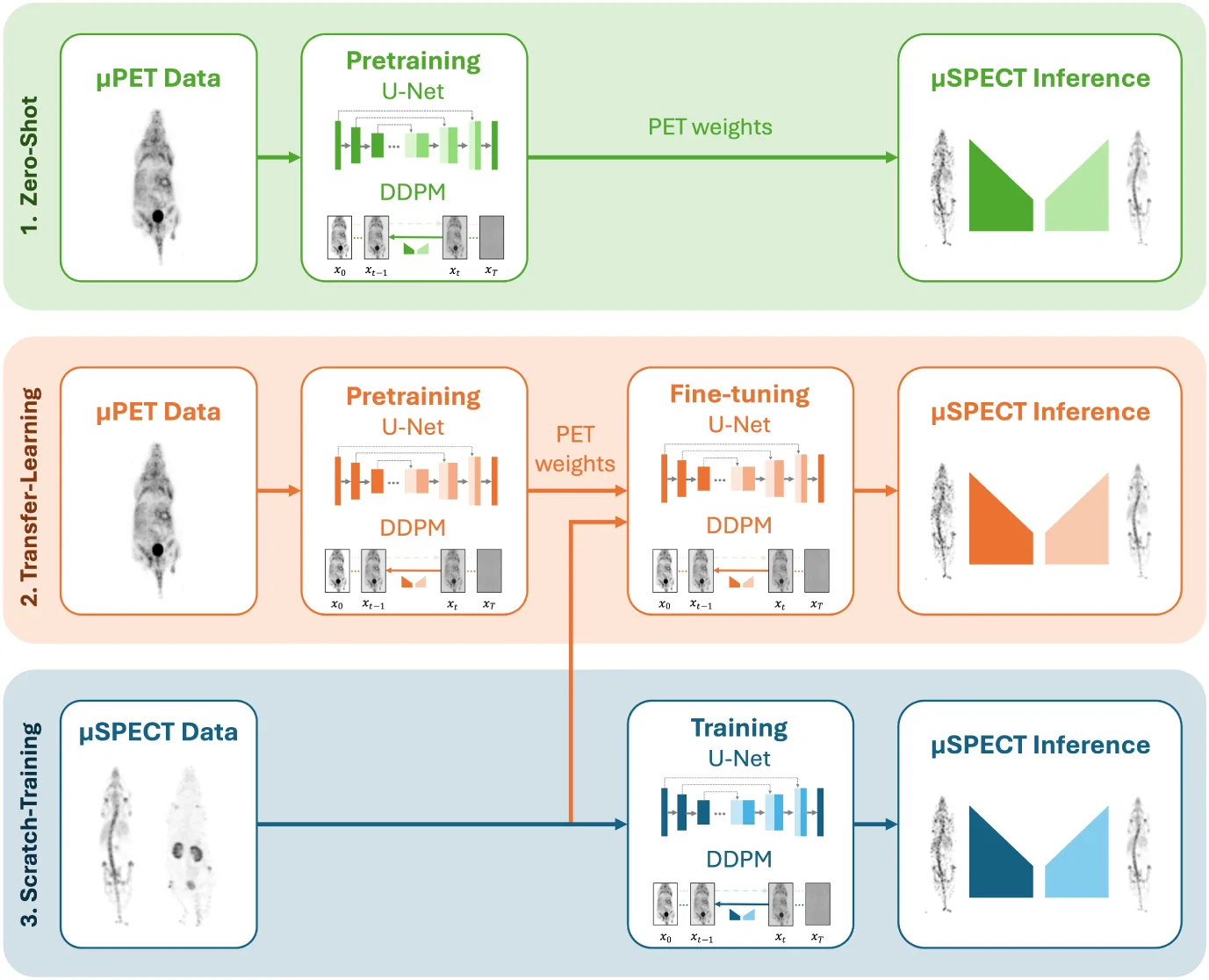
Overview of training strategies for U-Net and DDPM models. Both the U-Net and DDPM models were pretrained on μPET mouse data, prior to zero-shot evaluation and transfer-learning experiments.

## Materials and Methods

### μSPECT Data Acquisitions

All μSPECT images were acquired using the γ-CUBE system (Molecubes, Bruker Belgium SA). The scans were performed with a high-sensitivity collimator and a step-and-shoot protocol for total-body mouse imaging. Ten healthy mice were scanned in vivo using two tracers: ^99*m*^Tc-labeled dimercaptosuccinic acid (DMSA), which accumulates primarily in the kidney cortex,^32^ and ^99*m*^Tc-labeled hydroxydiphosphonate (HDP), which targets skeletal tissue.^33^ The acquisitions followed routine preclinical imaging protocols, without increased scan duration or administered activity to obtain higher-quality reference images. Injected activities were 13 MBq for ^99*m*^Tc-DMSA and 37 MBq for ^99*m*^Tc-HDP, in accordance with standard practice for renal and skeletal imaging, respectively. Each tracer was administered in separate sessions with sufficient clearance time between administrations. Scans were acquired at 15 min and 3 h postinjection for data augmentation, each with an acquisition time of 15 min, resulting in 40 image volumes (ten mice × two tracers × two time points). The data were split at the mouse level, such that all scans (both tracers and both time points) from one mouse were assigned to the test set, those from another mouse to the validation set, and the remaining eight mice to the training set. This yielded 32 training, four validation, and four test data sets. To expand the evaluation, an additional set of seven mice was scanned using ^99*m*^Tc-HDP at 3 h postinjection for 10 min and included exclusively in the test set, resulting in a total of 11 3D test images. A detailed overview of the data set composition is provided in Table 1. All animal experiments were conducted in accordance with institutional animal welfare regulations and were approved by the Ghent University Ethical Committee (ECD 22-42).

**Table 1:** Overview of the μSPECT Datasets Used for Training, Validation, and Testing

Tracer	Time Point	Duration	# Volumes	Split (Train/Val/Test)
DMSA	15 min	15 min	10	8/1/1
DMSA	3 h	15 min	10	8/1/1
HDP	15 min	15 min	10	8/1/1
HDP	3 h	15 min	10	8/1/1
HDP (extra)	3 h	10 min	7	0/0/7

All image reconstructions were performed using the maximum likelihood expectation maximization algorithm with 200 iterations, no subsets, and no postreconstruction filters. A 20% energy window centered around the 140 keV photopeak was used, and scatter correction was applied using the triple energy window method. No attenuation correction was used. Images were reconstructed with a 0.5 mm isotropic voxel size and subsequently converted to standardized uptake value (SUV) units. To simulate varying low-count levels, the original list-mode projection data were subsampled by randomly retaining 50%, 25%, and 10% of the detected events prior to reconstruction. This yielded per scan one high-count reference image and three corresponding low-count images.

### Training Strategy Experiments

Three training strategies were explored to evaluate the influence of modality transfer and prior knowledge on μSPECT denoising performance. An overview of these different training strategies is shown in Figure 2.

1 Zero-shot inference: to assess whether models pretrained on μPET generalize directly to μSPECT without any fine-tuning. This strategy represents direct cross-modality transfer of models and provides insight into how well μPET-derived feature representations capture the noise characteristics of μSPECT images.
2 Transfer-learning: to evaluate to what extent adapting pretrained μPET-based networks to μSPECT improves performance relative to zero-shot inference. In this approach, all network parameters were updated during fine-tuning, no layers were frozen. Full-network adaptation was selected to ensure that both low-level and higher-level feature representations can adjust to these modality-specific properties.
3 Training from scratch: to determine the performance of models trained solely on μSPECT data that can be considered as a reference. This strategy enabled assessment of whether training exclusively within target domain is superior, or whether μPET-based priors offer a benefit. It also allowed comparison of training convergence relative to transfer-learning.

### Deep Learning Models

This study evaluated two DL architectures: a 2D U-Net and a 2D DDPM, both adopted from prior PET denoising work. For each model, we retained the original network architecture and training setup as described in the respective PET implementations.

#### U-Net

The U-Net used here corresponds to the model proposed by Muller et al.^28^ for μPET denoising. It was trained on 28 ^18^F-fluorodeoxyglucose (FDG) μPET scans, acquired at a target injected activity of 8.15 MBq and reconstructed using ordered subsets expectation maximization (OSEM) with a voxel size of 400 μm. The training setup used paired data sets with mixed low-count levels of 50%, 25%, 10%, and 5% of the standard-count events. The model follows a conventional encoder–decoder design with four levels, skip connections, and a residual connection. It accepts three adjacent slices and predicts the denoised middle slice. The network contains about 12 million trainable parameters.

#### DDPM

The second architecture evaluated in this study is a 2D DDPM, originally introduced by Yu et al.^25^ for clinical PET denoising. For this study, the DDPM was trained from scratch on the same μPET mouse data set as described above for the U-Net. The network structure of the DDPM was kept the same as original; building upon a five-layer U-Net backbone, incorporating time step conditioning and residual blocks. A linear noise schedule with β-values ranging from 0.0001 to 0.02 over 1,000 diffusion steps is used. The model also takes three adjacent slices and denoises all three. For the final 3D volume, all predictions for a given slice are averaged to minimize the slice inconsistency effects seen in 2D diffusion models. This model has about 126 million trainable parameters.

### Training and Inference

#### Training Protocol

For all investigated cases, training was performed on μSPECT slices extracted in the coronal, sagittal, and transversal directions, yielding a total of 18,672 slices for the complete training set. Both the U-Net and DDPM models were implemented as 2D networks using a 2.5D strategy, where the networks process a 3-slice input consisting of the target slice alongside its two immediate adjacent neighbors (one on each side). This approach provides some spatial local context for noise reduction while maintaining computational efficiency. Furthermore, because photon noise is predominantly a local phenomenon, extending the receptive field to distant axial slices (i.e., fully 3D processing) was expected to offer diminishing returns, as distant information does not meaningfully inform the denoising of the target slice. A batch size of 64 was used for the U-Net and a batch size of 8 for the DDPM. During network optimization, both architectures used a fixed learning rate of 10^–4^ and MSE loss. The U-Net was optimized using the Adam optimizer, whereas the DDPM used the AdamW optimizer, consistent with their original implementations. For transfer-learning configurations, no freeze-unfreeze policy was employed, all network parameters across all layers were actively updated during fine-tuning to allow the full feature representation to adapt to the μSPECT domain. Both models trained from scratch and via transfer-learning were trained with early stopping applied when no improvement of the validation images was observed for 20 consecutive epochs.

#### Inference and Timing Measurements

During inference, denoising was performed slice-wise along all three orthogonal axes, after which the predictions were averaged voxel-wise to obtain the final volume. All training and inference on μSPECT images were performed on a single NVIDIA GeForce RTX 2080 Ti GPU (11,264 MiB memory). For the timing measurement protocol, training times were recorded from initiation of the script (incorporating data loading and model setup) until the completion of the best-performing validation epoch. The recorded training durations include the computational overhead of evaluating the validation set at intermediate epochs. For the DDPM, this validation process involves executing the full 1,000-time step reverse diffusion process per validation image. Inference times were calculated as the time required to load a single 3D image volume, perform slice-wise inference across all three anatomical directions, and average the resulting predictions to form the final 3D volume. The initial model loading and setup times were excluded from the inference measurements.

#### Normalization

For zero-shot inference, the pretrained models were applied directly, using the normalization procedures defined in their original implementations (mean-intensity normalization for the U-Net, scaling of the relevant intensity range to [0, 1] for the DDPM). For the models trained from scratch and those fine-tuned through transfer-learning, we used a bilinear intensity scaling strategy. μSPECT images acquired 15 min postinjection showed high-intensity bladder uptake, with values exceeding 100 SUV, while the relevant uptake range elsewhere in the body was confined to 10 SUV. To prevent the networks from overfitting to this outlier region, intensities above 10 SUV were compressed into a dynamic range of 1 SUV using a forward transformation *f*(*x*):

$$f\left(x\right)=\{\begin{array}{ll} x, & x\leq 10\\ 10+\alpha \left(x-10\right), & x>10 \end{array}$$

where $\alpha =\frac{1}{x_{\max}-10}$ and *x*_max_ is the maximum intensity in the image. This transformation results in images with an effective range of [0, 11] SUV. Subsequently, architecture-specific normalizations were applied for network training and inference: images for the DDPM were linearly scaled to the [0, 1] range, whereas images for the U-Net underwent mean-intensity normalization. To ensure accurate quantitative evaluation, the network outputs were first linearly rescaled back to the [0, 11] SUV range by reversing their respective architecture-specific normalizations. Following this step, the inverse transformation *f* ^–1^(*y*) was applied to map the values back to the original, uncompressed SUV space prior to bias calculations:

$$f^{-1}\left(y\right)=\{\begin{array}{ll} y, & y\leq 10\\ 10+\frac{1}{\alpha }\left(y-10\right), & y>10 \end{array}$$

### Data Efficiency Experiments

In addition, all strategies were evaluated with varying amounts of μSPECT data to examine how the relative performance of the different training strategies changes as a function of available training data. Models were trained on progressively larger subsets of the data set, corresponding to one mouse (12.5%), two mice (25%), four mice (50%), six mice (75%), and eight mice (100%) of the training set. The same subset of animals was used for both the U-Net and the DDPM to ensure a consistent comparison between models.

### Performance Evaluation

To assess the accuracy of quantitative uptake measurements, SUV measures were extracted from volumes of interest (VOIs) and compared to the high-count reference. For the DMSA test image acquired 3 h postinjection, 12 spherical VOIs were placed in the renal cortex as shown in Figure 7. Over these VOIs, mean and maximum SUV (respectively SUV_mean_ and SUV_max_) were extracted from both the low-count and denoised images, and absolute bias with respect to the high-count was calculated. In addition to these localized VOI measurements, voxel-wise absolute SUV bias was analyzed within skeletal segmentations in the 3 h postinjection HDP scans. Quantitative image quality was assessed using the root mean squared error (RMSE) and structural similarity index measure (SSIM) relative to the high-count reference. For SSIM, relative improvements were defined in terms of the reduction in its distance to perfect similarity (1-SSIM).

Statistical comparisons were performed for the quantitative image quality metrics and SUV measures. For each model, denoised outputs were compared with the corresponding low-count images, and performance differences between the three training strategies (zero-shot, transfer-learning, and training from scratch) were also evaluated. Normality of paired differences was verified using the Shapiro-Wilk test. When normality was satisfied, two-tailed paired *t* tests were applied. Otherwise, the nonparametric Wilcoxon signed-rank test was used. P-values below 0.05 were considered statistically significant.

To further evaluate the performance of the three training strategies and determine whether μPET-pretraining captures meaningful cross-modality features, additional evaluations were performed using the Molecubes Quality Control (QC) phantom. This phantom includes a uniform section for assessing noise suppression and a section with contrast spheres to evaluate contrast and edge preservation. These results are provided in the Supporting Information.

## Results

### Zero-Shot Denoising Performance of μPET-Pretrained Models on μSPECT

Figure 3 displays maximum intensity projection (MIP) images of a mouse ^99*m*^Tc-HDP scan acquired 3 h postinjection. The high-count reference, acquired with an injected activity of 37 MBq, is shown together with the corresponding low-count inputs at 10%, 25%, and 50% of the detected counts. For each count level, the figure includes the low-count input and the outputs obtained for zero-shot inference of the μPET-pretrained U-Net and DDPM. Across all evaluated levels, the U-Net visibly reduces the noise compared to low-count inputs while preserving functional structure, including vertebral uptake patterns. Residual speckle-like noise is still present, particularly at lower count levels. The DDPM outputs appear visually smoother and more noise-free across all count levels. They are highly similar between levels, with only minor increases in retention of fine detail at higher counts. However, fine patterns such as the spine appear slightly less sharply defined than in the U-Net outputs and low-count images. At 10%, both the U-Net and DDPM introduce a hotspot in the liver, as indicated with a blue arrow in Figure 3.

**Figure 3:**
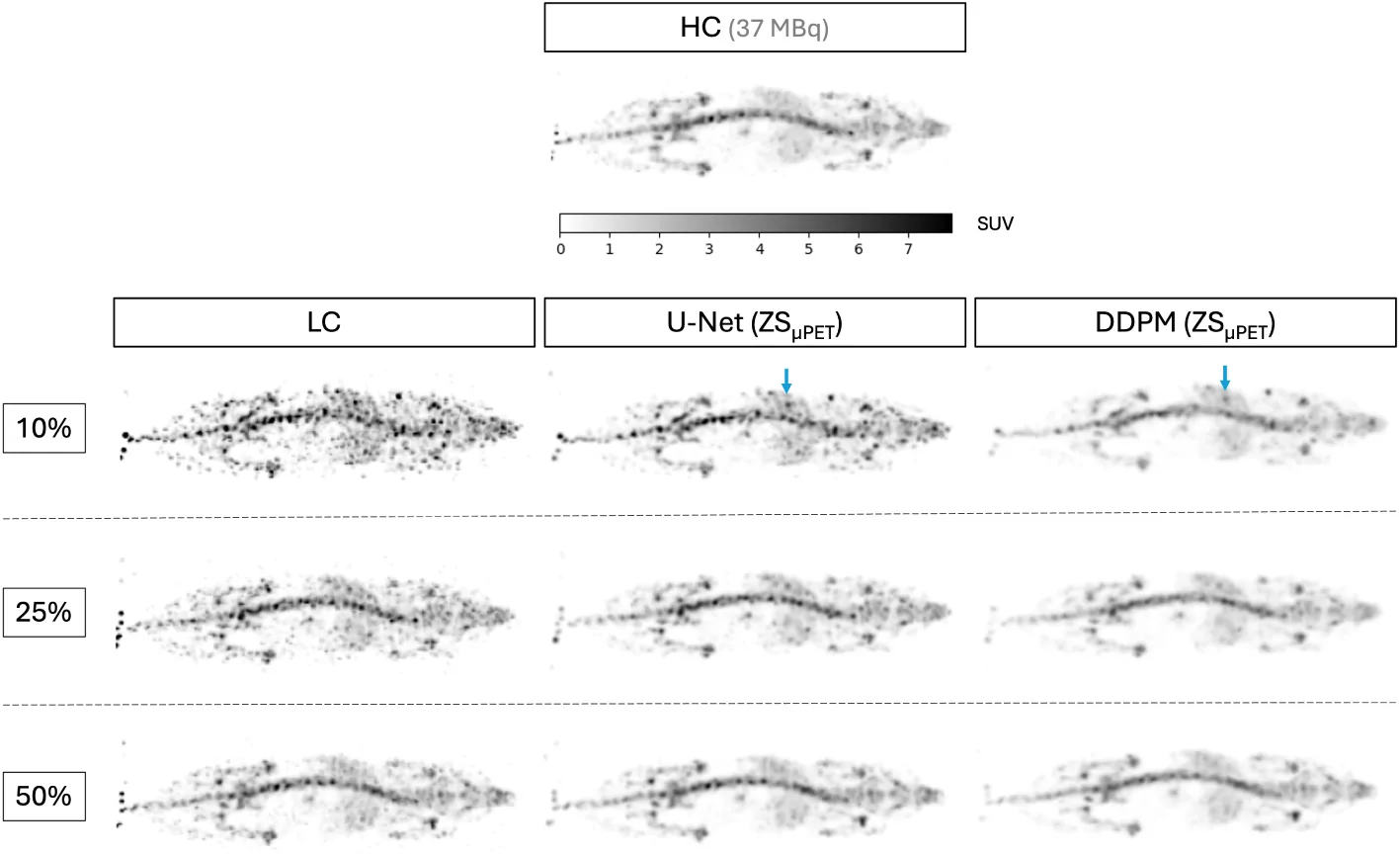
Maximum intensity projection images of a mouse ^99*m*^Tc-HDP scan acquired 3 h postinjection. The high-count (HC) reference (injected activity: 37 MBq) is shown together with the corresponding low-count (LC) inputs at 10%, 25%, and 50% and the zero-shot (ZS_μPET_) U-Net and DDPM images. The blue arrow indicates a hallucination in the liver.

Figure 4 summarizes the zero-shot denoising performance using RMSE and SSIM calculated over the entire 3D test images, complemented by slice-wise metric distributions. The U-Net consistently improves both metrics across all evaluated count levels. The DDPM shows very strong performance at the lower count levels, outperforming the U-Net in both RMSE and SSIM. At the 50% count level, the U-Net model performs slightly better and the improvement of DDPM denoising over the low-count images becomes smaller. Across methods, the slice-wise metric distributions generally become more compressed as photon statistics increase, reflecting reduced intravolume variability at higher count levels.

**Figure 4:**
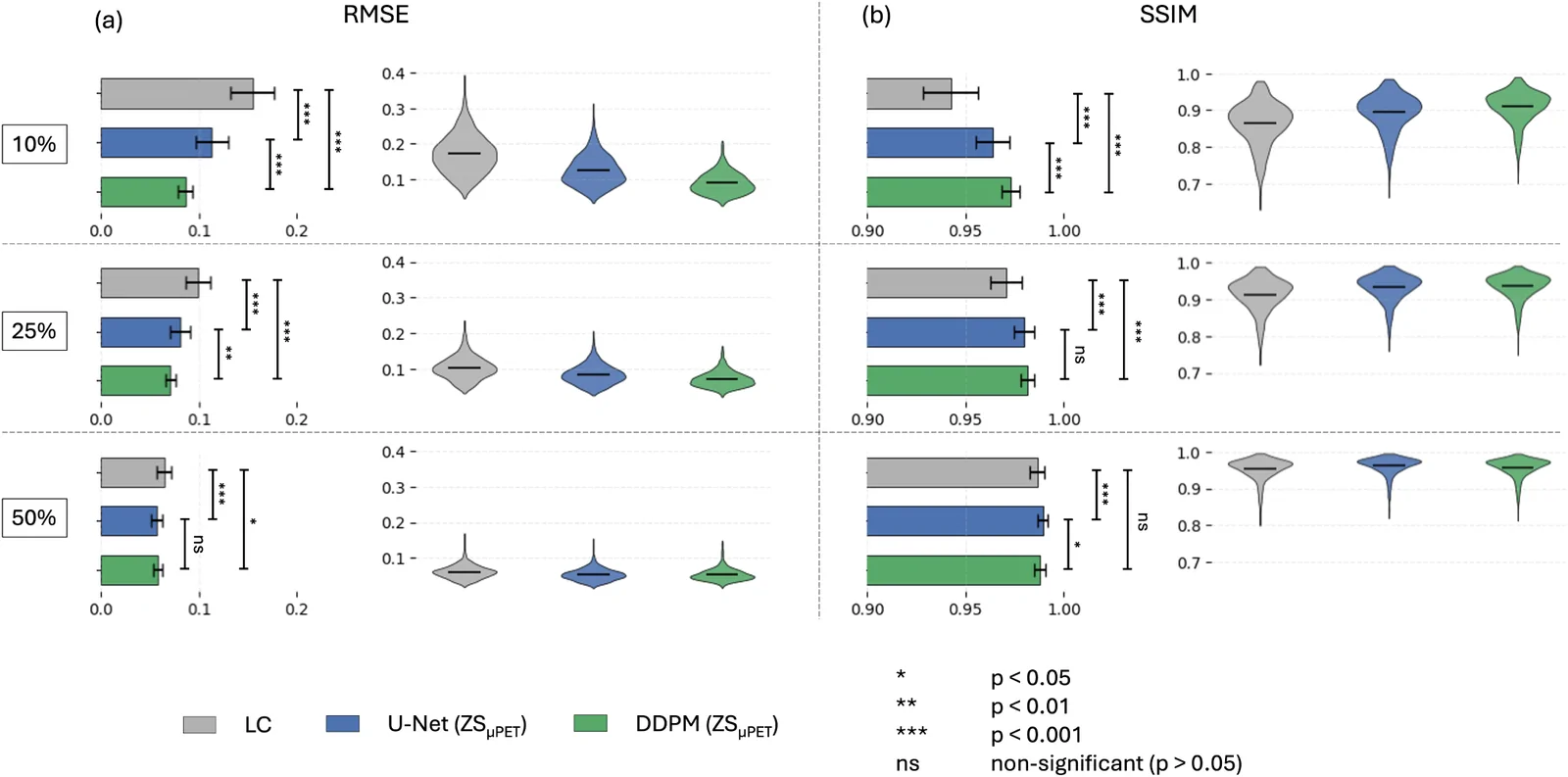
Bar plots show volume-level (a) RMSE and (b) SSIM for low-count (LC) images and zero-shot (ZS_μPET_) U-Net and DDPM reconstructions at 10%, 25%, and 50% count levels, computed across the 11 3D test volumes, with statistical significance assessed at volume level. Violin plots show the corresponding slice-wise (a) RMSE and (b) SSIM distributions, computed per axial slice across all 3D test volumes, highlighting intravolume variability.

### Performance Comparison of Zero-Shot, Transfer-Learned, and Scratch-Trained Models

Table 2 reports the training and inference times for the zero-shot, transfer-learned, and scratch-trained models. U-Net models exhibit substantially shorter training and inference times compared to DDPMs. In addition, transfer-learning markedly reduces training time relative to training from scratch for DDPMs. Representative training and validation learning curves for both architectures are provided in Supplementary Figure S1.

**Table 2:** Training Time, Number of Epochs, and Inference Time for All Training Strategies for the 2D U-Net and 2D DDPM^a^

Model	Training	Epochs	Inference
U-Net (ZS_μPET_)	-	-	2 min
U-Net (TL_μPET_)	1 h	32	2 min
U-Net (Scratch)	1 h	49	2 min
DDPM (ZS_μPET_)	-	-	2 h
DDPM (TL_μPET_)	14 h	23	2 h
DDPM (Scratch)	44 h	76	2 h

a Inference time corresponds to the reconstruction of one full 3D volume, with the DDPM using 1,000 reverse diffusion timesteps.

Figure 5 displays the MIP images for the same scan as shown in Figure 3, now including all evaluated training strategies: zero-shot, transfer-learning and training from scratch. For the U-Net models, both transfer-learning and scratch-training lead to a clear improvement over the zero-shot baseline, yielding visually very similar images. Speckle-like noise is effectively suppressed, while the main functional structures are preserved. For the DDPMs, both transfer-learning and scratch-training also slightly improve the visual appearance relative to the zero-shot output, again resulting in images that are highly similar. However, the images show some loss of fine functional detail, particularly within the spinal uptake pattern when compared to the high-count reference. At the 10% count level, the hotspot in the liver observed for the zero-shot models is suppressed.

**Figure 5:**
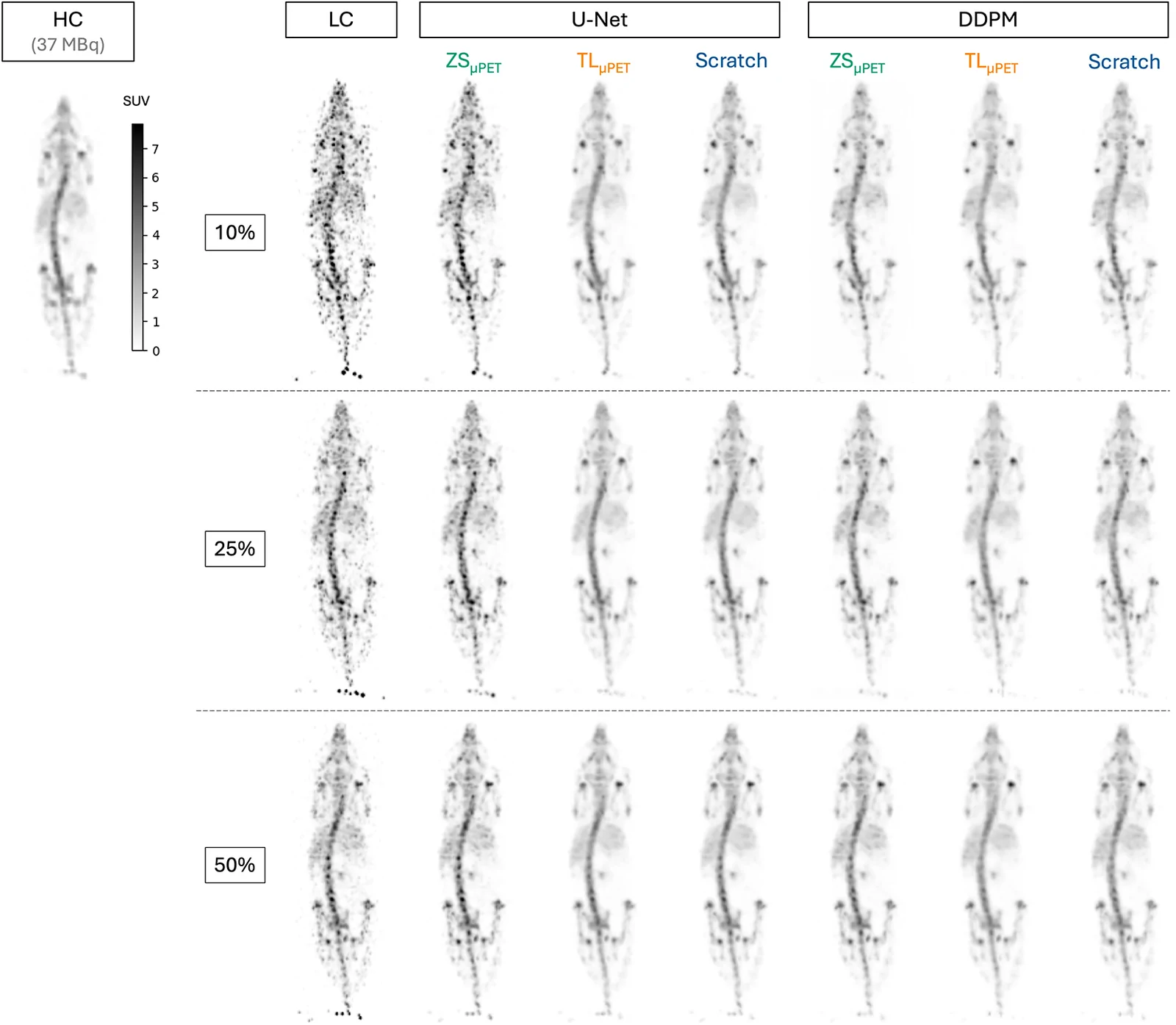
Maximum intensity projection images of a mouse ^99*m*^Tc-HDP scan acquired 3 h postinjection. The high-count (HC) reference (injected activity: 37 MBq) is shown together with the corresponding low-count (LC) inputs at 10%, 25%, and 50% and the zero-shot (ZS_μPET_), transfer-learned (TL_μPET_), and scratch-trained (Scratch) U-Net and DDPM images.

Figure 6 presents the volume-level RMSE and SSIM for both model architectures across the different training strategies. For the U-Net models, both transfer-learned and scratch-trained models perform significantly better than the zero-shot baseline for most count-levels and both metrics. An exception is observed at the 50% count level, where zero-shot SSIM performance is comparable to or better than the trained models. When directly comparing the trained variants, the scratch-trained U-Net significantly outperforms the transfer-learned model, with the exception of RMSE at 10% where no significant difference is observed. For the DDPM architecture, both transfer-learned and scratch-trained models only significantly outperform the zero-shot baseline at the lowest count level. Training from scratch yields better performance than transfer-learning at the 25% and 50% count levels, whereas transfer-learning performs better at the lowest count level. Significance with respect to the low-count images is omitted from Figure 6 for clarity. Both transfer-learned and scratch-trained models significantly outperform the low-count images across nearly all conditions, with the exception of the transfer-learned DDPM at the 50% count level.

**Figure 6:**
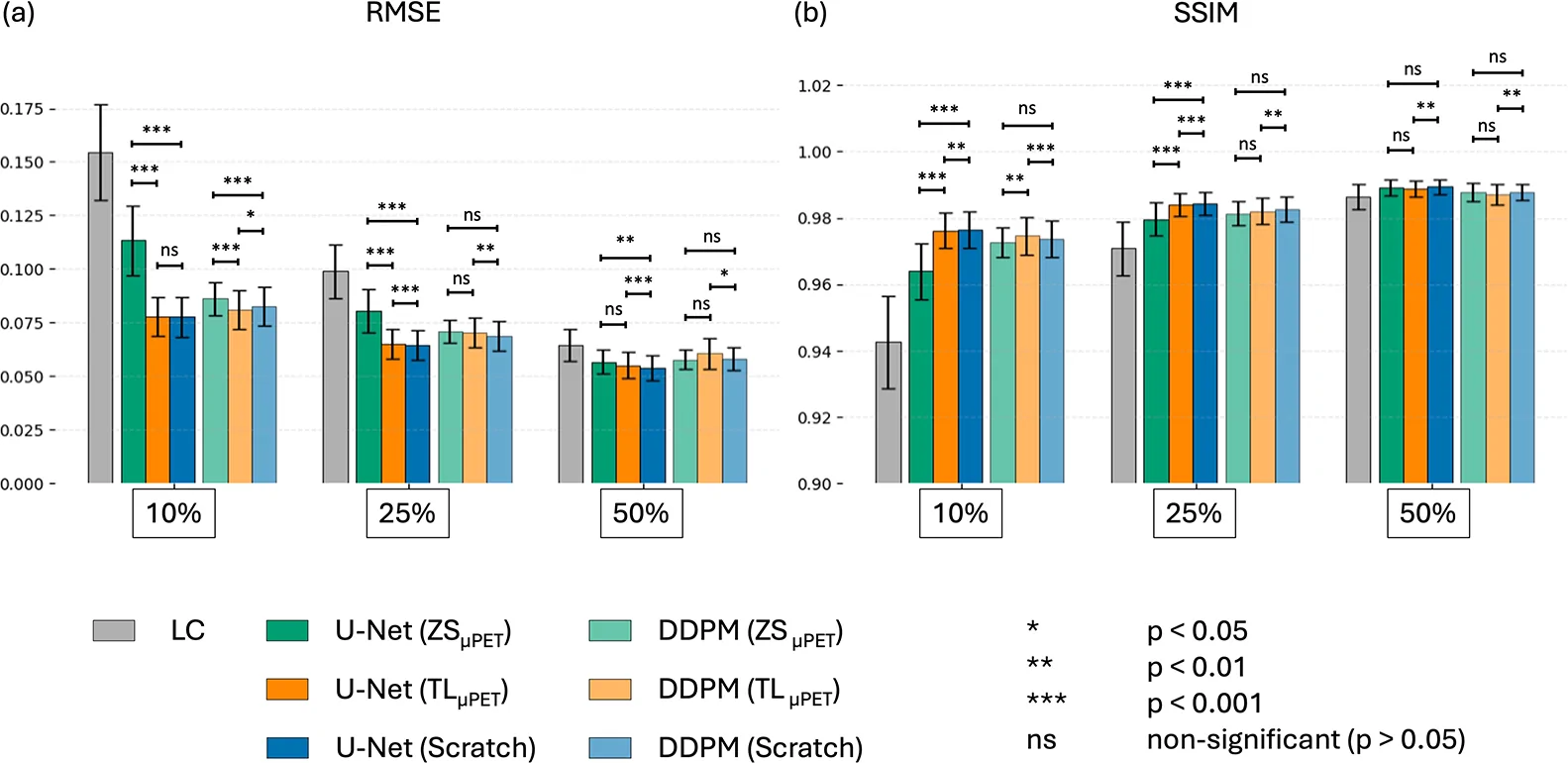
Volume-level (a) RMSE and (b) SSIM for low-count (LC) images and the corresponding U-Net and DDPM denoised outputs at 10%, 25%, and 50% count levels, computed across 11 test volumes. Each bar represents the mean metric value over subjects, with error bars indicating the standard deviation. Pairwise statistical comparisons between methods are reported for each count level.

The qualitative evaluation of SUV_mean_ and SUV_max_ bias within VOIs in the renal cortex revealed consistent performance trends across both architectures (Figure 7). While the zero-shot models achieved a measurable reduction in bias compared to the low-count baseline, both transfer-learning and scratch-training strategies provided superior quantitative recovery. The relative benefit of these domain-specific training strategies was most pronounced at lower count levels and gradually lowered at higher count statistics. Between the two training approaches, transfer-learning consistently yielded the lowest average SUV_mean_ and SUV_max_ biases. However, these gains over scratch-training were generally not statistically significant. For clarity, statistical significance relative to the low-count baseline is not shown.

**Figure 7:**
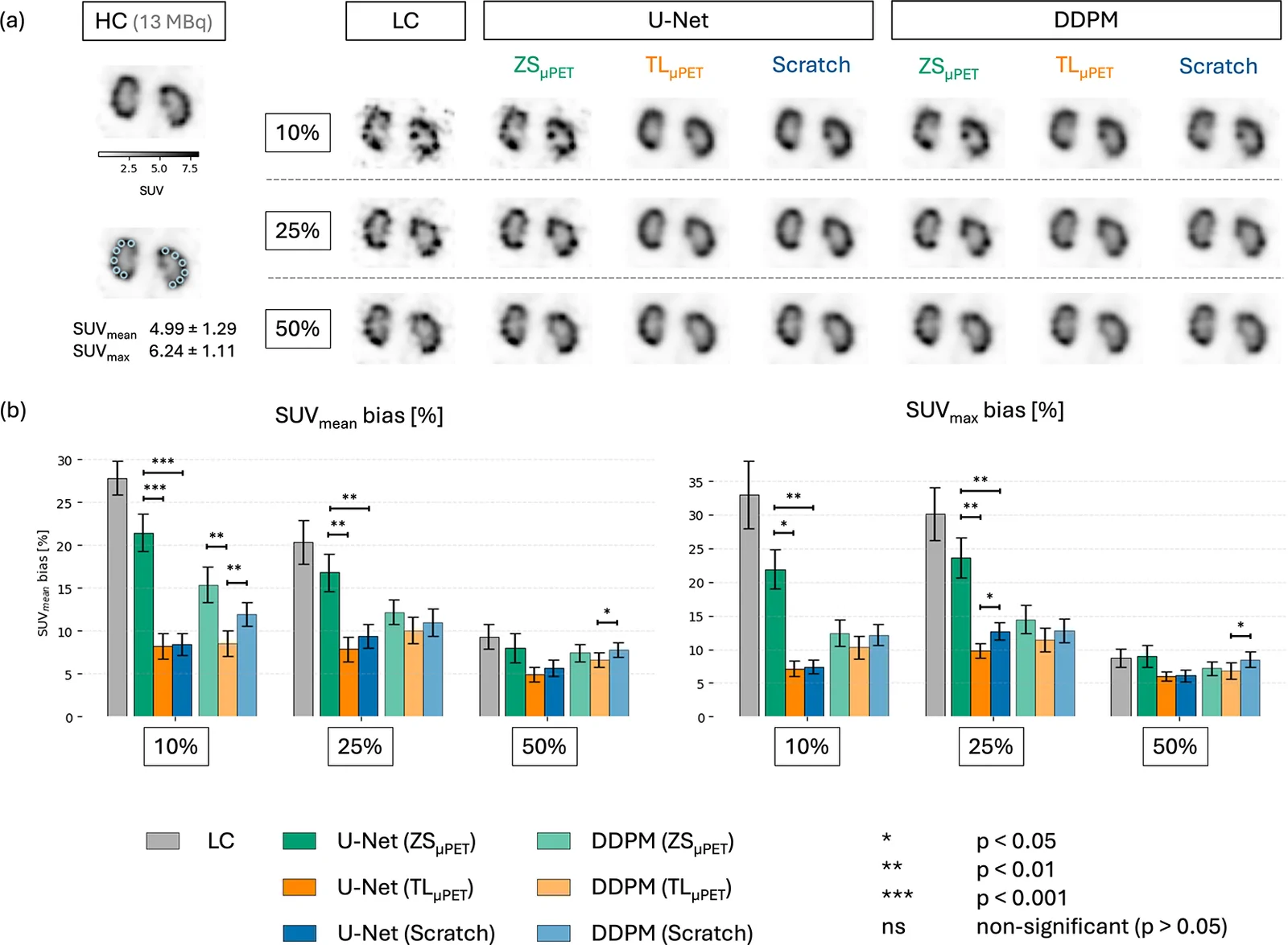
(a) Representative kidney slice for the high-count (HC) reference, low-count (LC) input, and all U-Net and DDPM denoising configurations at 10%, 25%, and 50% count levels. (b) Absolute SUV_mean_ and (c) SUV_max_ bias for 12 spherical VOIs placed in the kidney cortex of a ^99*m*^Tc-DMSA scan acquired 3 h postinjection. Bars represent the mean of the absolute bias values across the 12 VOIs, and error bars denote the standard deviation (divided by a factor of 5 for visualization purposes). Pairwise statistical comparisons between methods are reported for each count level.

Figure 8 shows voxel-wise SUV bias distributions within skeletal uptake, reporting for each bias value the fraction of skeletal voxels within that threshold. Across both model architectures, the bias distributions for different models converge with increasing count level. For the U-Net architecture, both transfer-learned and scratch-trained models outperform the zero-shot model and show largely overlapping distribution at lower count levels. With increasing counts, these trained model curves slightly diverge and scratch-training performs best, while the gap with the zero-shot model gets smaller. This behavior is consistent with the trends observed in the image quality metrics (Figure 6). For the DDPMs, the curves for all three training strategies lie much closer together, indicating smaller performance differences in skeletal SUV bias between the methods. While the distributions largely overlap across all count levels, subtle shifts in their relative ordering occur as photon statistics increase. At the 10% count level, transfer-learning performs slightly better than the other approaches, followed by scratch-training. At 25%, performance becomes highly similar across all three strategies, and by the 50% count level, the zero-shot model achieves the best performance while transfer-learning performs slightly worse.

**Figure 8:**
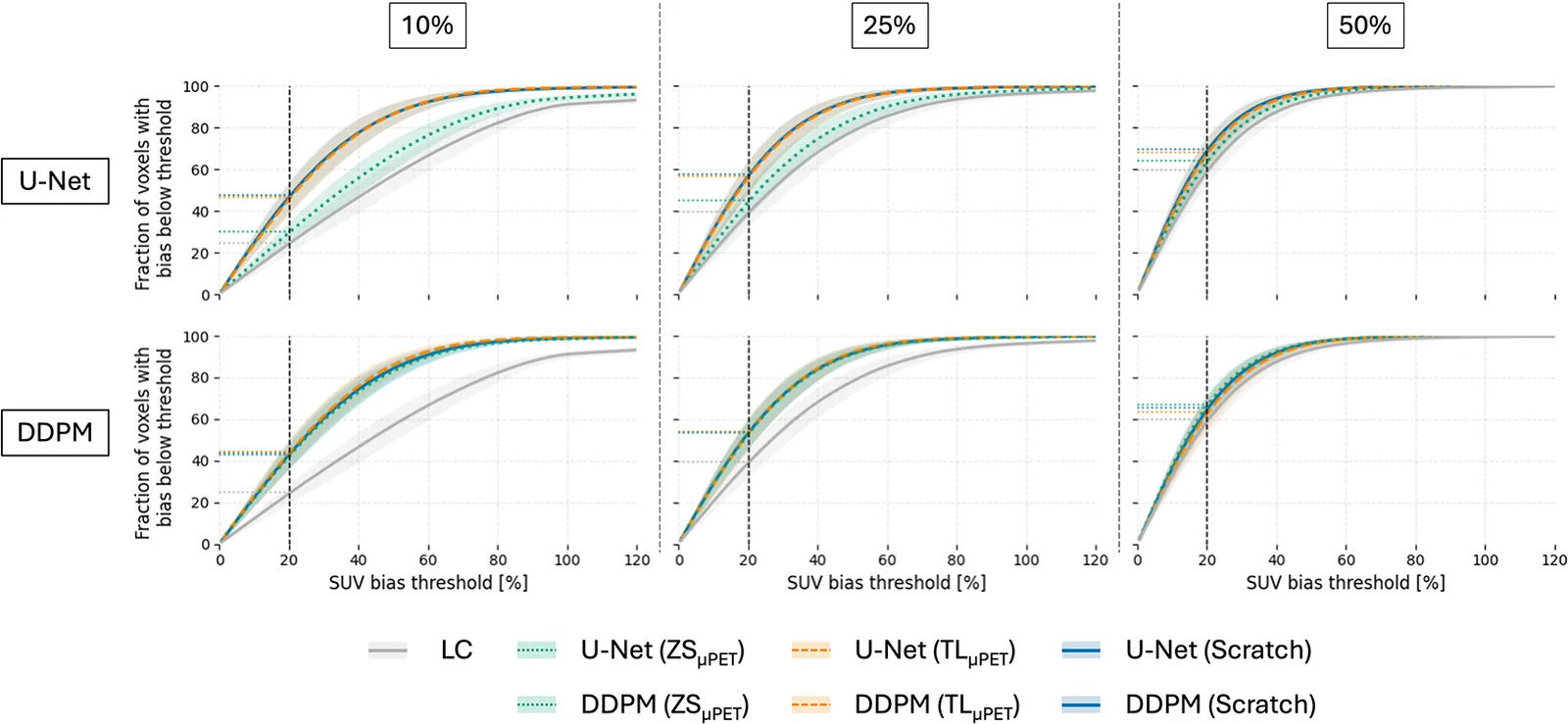
Cumulative voxel-wise SUV bias curves for U-Net and DDPM at different count levels, showing the fraction of skeletal voxels whose absolute SUV bias from the high-count reference is below a given threshold. Curves represent the mean across eight ^99*m*^Tc-HDP test volumes acquired 3 h postinjection, with shaded bands indicating the corresponding 95% confidence intervals.

### Influence of Data Set Size

Figure 9 illustrates the effect of training data set size on denoising performance at the 10% count level, reporting volume-level RMSE and SSIM as a function of the fraction of μSPECT training data used. For both model architectures, transfer-learning yields superior performance at lower training data fractions, achieving lower RMSE and higher SSIM compared to training from scratch. As the amount of training data increases, the performance gap between transfer-learning and scratch-training decreases. However, the convergence behavior differs between the two architectures. For the U-Net, scratch-training becomes increasingly competitive as data scales, ultimately matching or even exceeding transfer-learning performance at the 100% data fraction. Conversely, for the DDPM, the zero-shot variant demonstrates a high baseline performance, due to which the transfer-learning curves show a less steep improvement than other curves. While scratch-training rapidly improves to become comparable at intermediate data volumes, transfer-learning ultimately retains a statistically significant advantage when 100% of training data is used.

**Figure 9:**
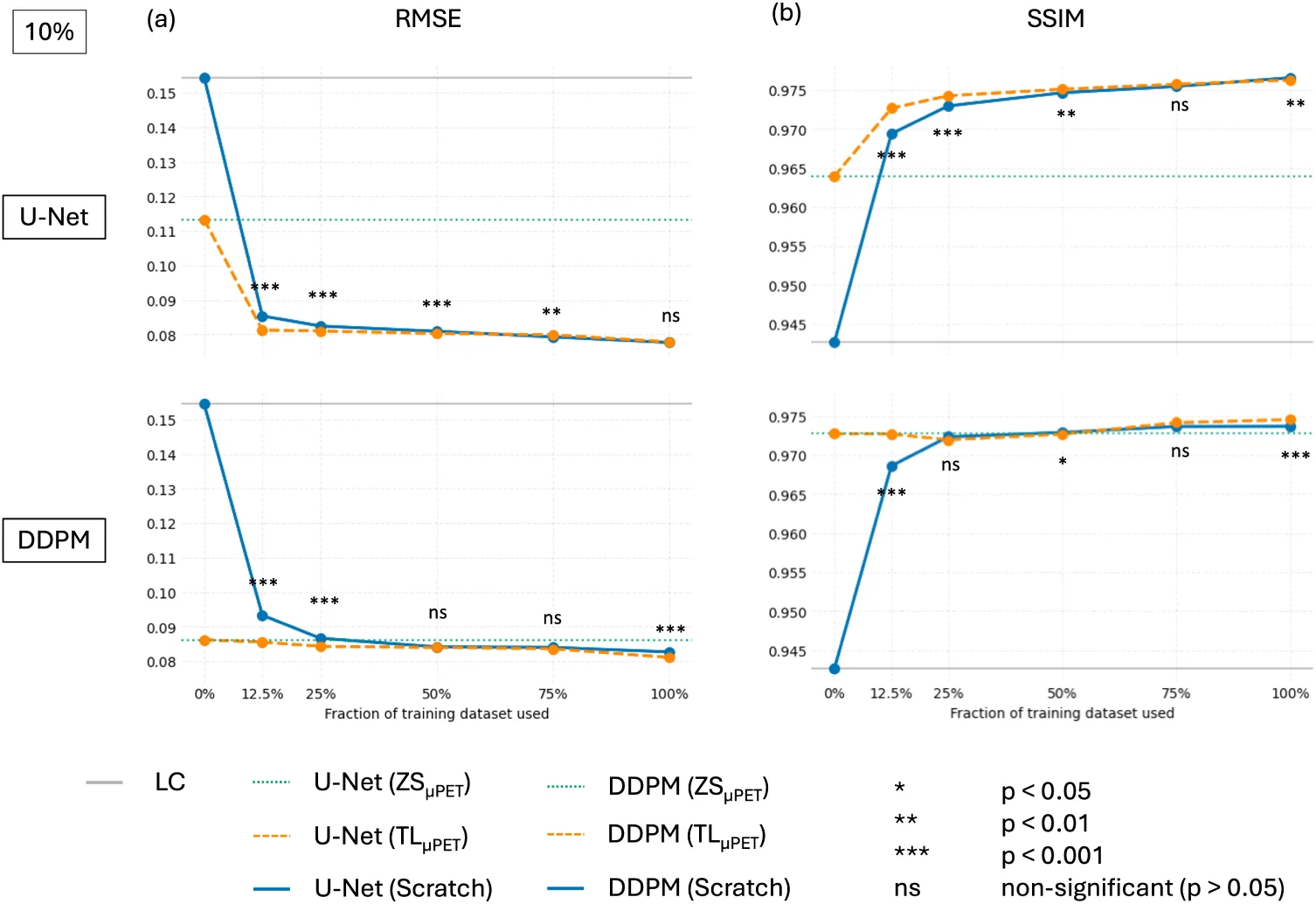
(a) RMSE and (b) SSIM as a function of the fraction of training data used for U-Net and DDPM denoising models at the 10% count level. Curves show mean performance across all test volumes, with comparison to low-count (LC) and zero-shot PET-pretrained baselines (ZS_μPET_). Pairwise statistical comparisons between transfer-learned and scratch-trained models are reported for each training data set size.

## Discussion

### Rationale for Domain Adaptation in μSPECT

This study examined the use of DL-based denoising to enable dose reduction in μSPECT imaging. Two representative 2D architectures, a U-Net and a DDPM, were evaluated across multiple count levels corresponding to 10%, 25%, and 50% of standard counts. This study is motivated by the imbalance in the DL-based denoising literature, which predominantly targets clinical imaging, while preclinical applications remain underrepresented. Preclinical studies are further challenged by limited data set sizes, which constrain the direct training of data-intensive DL models. To address this data limitation in DL, transfer-learning is sometimes employed to reuse feature representations learned during pretraining on a source domain, thereby reducing the amount of target domain data required.^34^ This strategy is increasingly researched in medical contexts,^35^ where small data sets are common in the context of annotations which are time-consuming and expensive. Notably, transfer-learning studies often extend beyond related medical data and leverage large natural image data sets such as ImageNet.^36^ However, prior work has shown that transferability generally improves as the source and target domains, as well as the learning tasks, become more closely aligned.^34^ In the context of μSPECT denoising, PET constitutes a particularly relevant source domain, as both modalities are molecular imaging techniques that rely on radiotracer distribution. The principle of transferring PET-trained models to the SPECT domain has previously been explored by Ni et al.^37,38^ in the context of Alzheimer’s disease detection. The close relationship between PET and SPECT is further supported by studies that investigate direct translation between the two modalities.^39–42^ In this study, we therefore considered models pretrained on μPET (mouse) data. Building on these observations, we investigated three training strategies: (1) zero-shot application of μPET-pretrained models, (2) transfer-learning of these models to the μSPECT domain, and (3) training from scratch. In addition, the influence of training data set size on the relative performance of these approaches was assessed.

### Comparison of Training Strategies

Both the U-Net and DDPM achieved meaningful denoising performance in a zero-shot setting, indicating that μPET-pretrained networks successfully learn feature representations that generalize beyond their source modality. Despite no prior exposure to μSPECT data, both architectures were able to suppress noise while preserving large-scale tracer uptake patterns. At the lowest count level, zero-shot models reduced RMSE on average by more than 25% and improved SSIM by more than 35%. However, the U-Net showed some residual noise, particularly at the lower count levels. Furthermore, both models occasionally produced localized artifacts due to insufficient noise suppression, manifesting as hotspots that could be misinterpreted. These effects reflect the inability of zero-shot models to fully capture modality-specific noise characteristics. This limitation is expected given the fundamental differences between PET and SPECT imaging, such as photon energy, detector design, and the use of collimation, which directly influence noise and resolution.

Transfer-learning substantially mitigated these denoising artifacts resulting from the domain gap. Fine-tuning on limited μSPECT data improved noise suppression for U-Net and enhanced functional fidelity for both models. The largest improvements were observed at the lowest count level. At this level, transfer-learned U-Net models achieved mean improvements exceeding 30% in both RMSE and SSIM relative to their zero-shot counterparts. For the DDPM architecture, the mean improvement was approximately 5% for RMSE and SSIM, reflecting that the zero-shot baseline already exhibited strong performance. In addition to improvements in global image quality metrics, transfer-learning yielded more accurate quantitative uptake estimates within regions of interest compared to zero-shot inference, indicating that domain adaptation also benefits regional activity quantification. The benefit of transfer-learning compared to scratch-training was most pronounced when training data were scarce. As the μSPECT data set size increased, training from scratch became increasingly competitive, with the maximal performance advantage of transfer-learning decreasing from over 10% to below 3% in RMSE, and from over 15% to below 6% in SSIM. These trends are consistent with prior studies comparing transfer-learning and scratch-training in general medical imaging tasks, which report that transfer-learning yields comparable or superior performance, particularly in low-data regimes.^35,43,44^ The study of Tajbaksh et al.^45^ additionally confirms that as data set size increases, the relative benefit of transfer-learning diminishes. Moreover, they also report that transfer-learning facilitates faster convergence during training on the target domain. Beyond these advantages, transfer-learning has also been reported to mitigate overfitting and improve generalizability when only limited training data are available,^34,45^ although this was not explicitly evaluated in the present study. From a practical perspective, these findings highlight a trade-off between performance and data availability. Zero-shot inference provides immediate applicability, but remains sensitive to source-target domain mismatches. Training from scratch can yield strong performance when sufficient data are available, but is often impractical in small animal settings. Transfer-learning emerges as a robust and data efficient strategy.

To verify that benefits of transfer-learning stem from meaningful feature adaptation rather than incidental optimization effects, model performance was evaluated on the Molecubes QC phantom (Supplementary Figure S2). In the zero-shot setting, the μPET-pretrained models successfully suppressed a substantial amount of noise in the uniform region while simultaneously maintaining the definition of fine rods and edges. This indicates that the pretraining process on the μPET domain allowed the models to learn representations of molecular imaging noise that generalize to the μSPECT modality. While both transfer-learning and scratch-training further improved noise suppression by tuning to the specific characteristics of μSPECT data, the transfer-learned outputs retained distinct visual characteristics that distinguish them from scratch-trained results. This difference suggests that transfer-learning does not simply overwrite the source domain information or merely act as an alternative parameter initialization. Instead, the final model parameters remained influenced by the feature set acquired during pretraining. These observations are further supported by the training and validation curves in Supplementary Figure S1, which show that transfer-learned models initialize at a substantially lower loss than scratch-trained models.

### Architectural Comparison

When comparing the two architectures directly in zero-shot setting, the DDPM demonstrated significantly superior noise suppression and recovery of quantitative uptake in regions of interest compared to the U-Net, particularly at the lower count levels. This performance gap reflects the DDPM’s ability to learn more robust distributions of noise, allowing it to better generalize to the μSPECT domain. However, when comparing the architectures after transfer-learning or scratch-training, the U-Net and DDPM achieved visually comparable denoising performance. Some subtle differences included the slightly better preservation of small uptake patterns for the U-Net models. Despite the substantially higher computational burden and parameter count of the DDPM, the transfer-learned and scratch-trained images were not consistently superior to those obtained with the U-Net. This contrasts with findings reported in clinical PET, CT, and SPECT studies, where diffusion-based models have demonstrated clearer performance advantages.^20,24–26,46,47^ Future investigations will focus on identifying the potential causes of this lower performance on preclinical SPECT data sets and exploring methods to improve the unfavorable time-quality trade-off of the DDPMs. To mitigate the lengthy inference times characteristic of standard DDPMs, accelerated deterministic samplers such as denoising diffusion implicit models can be used to achieve comparable image quality in significantly fewer inference steps.^48^ Furthermore, computational efficiency could be enhanced through partial diffusion frameworks,^49^ which initiate the reverse process from the low-count image at an intermediate time step rather than from complete Gaussian noise, or through the application of latent diffusion models that perform the generative process in a compressed, lower-dimensional space.^50^

### Influence of the Source Domain

To further investigate the influence of source domain alignment, the potential of transfer-learning from the clinical (human) PET domain to preclinical SPECT was evaluated. Clinical data sets are often significantly larger and more diverse than preclinical ones, offering a potentially richer feature set for pretraining. Additionally, the prevalence of DL studies in clinical modalities has produced a larger selection of pretrained architectures that can readily be adapted, which is currently far less developed in the preclinical domain. While this shift from human to rodent data introduces an additional domain gap, cross-species transfer-learning has been shown to be feasible in prior work, such as the cross-species brain extraction tasks demonstrated by Yu et al.^51^

This evaluation used the DDPM model previously trained on 317 ^18^F-FDG total-body PET scans from the original implementation by Yu et al.^25^ for clinical PET denoising. In a zero-shot setting (Supplementary Figure S3), this clinical model demonstrated excellent noise suppression, providing visually noise-free images. However, it exhibited reduced retention of functional detail and substantial anatomical distortions, most notably in the spine. These artifacts likely stem from the more significant anatomical gap between species. This is further reflected in the quantitative metrics, where the μPET-pretrained models achieved superior RMSE and SSIM compared to the clinical PET-pretrained version (Supplementary Figure S4).

### Practical Applications

Beyond methodological insights, the proposed DL-based denoising framework has clear practical relevance for preclinical research. It can be used to lower administered activities in diagnostic scans, thereby reducing radiation burden to the animals. In addition, it enables shorter acquisition times to increase experimental throughput and it can improve image quality in situations where low count statistics are unavoidable. In theranostic studies, accurate dosimetry requires longitudinal imaging over extended time periods, often spanning several days postinjection.^52^ This is needed to quantify organ-specific uptake to determine maximal therapeutic activity levels in the context of personalized medicine. At later time points, radioactive decay and biological excretion substantially reduce count statistics, resulting in severely degraded image quality. Improved denoising performance is also relevant for dynamic imaging. These studies benefit from very short temporal frames to capture tracer kinetics, but are inherently constrained by limited photon statistics. Another application area where the denoising framework can have substantial impact is imaging of tracers with intrinsically low photon yield, such as ^177^Lu, for which only a small fraction of decays result in detectable gamma emissions.^53^ Similar challenges arise for alpha-emitting radionuclides used in targeted alpha therapy, where low administered activities lead to limited precision in activity estimation, as in the case of ^223^Ra or ^225^Ac. For these radionuclides, quantitative SPECT imaging is further complicated by complex decay schemes and the reliance on gamma emissions from daughter isotopes.^54^ Comparable low-count statistics have also been reported for PET imaging of radionuclides such as ^90^Y, which has an extremely low positron yield,^55^ and ^89^Zr, which is typically imaged at late time points and where DL-based denoising has been shown to improve image quality and quantitative reliability.^56,57^ Across these contexts, DL-based denoising is a practical means to alleviate the fundamental limitations imposed by low photon statistics, thereby expanding the feasibility and quantitative reliability of preclinical SPECT studies.

### Study Limitations

Several limitations should be considered when interpreting the findings of this study. First, the available data set was limited and acquired on a single μSPECT system. Although two different tracers and two acquisition time points were included to improve generalizability, the results may not directly extend to different scanners, collimators, reconstruction settings, or tracers. Furthermore, the reference images corresponded to standard dose acquisitions rather than noise-free ground-truth images. Low-count images were generated through random count subsampling instead of independent low-dose acquisitions, which may not fully capture all physical effects associated with true low-dose imaging. Additionally, the reliance on a 2D sampling strategy for 3D μSPECT volumes represents a methodological trade-off.

## Conclusions

The results show that DL-based denoising enables effective dose reduction in μSPECT imaging while preserving quantitative accuracy. PET-pretrained U-Net and DDPM models demonstrate meaningful zero-shot performance, and further improvements are obtained through transfer-learning on μSPECT data. The effectiveness of zero-shot inference and transfer-learning depends on the degree of similarity between the source and target domains: models pretrained on μPET data provide more relevant prior information than those pretrained on clinical PET. Transfer-learning is particularly beneficial when training data sets are small, whereas training from scratch becomes competitive or even superior as more data becomes available. For the evaluated setup, the U-Net provides the most robust performance with lower computational cost, while the DDPM offers no consistent advantage despite its higher complexity, which deserves further investigations. These findings indicate that DL-based denoising via transfer-learning from PET, is a practical strategy for low-dose μSPECT imaging, while directly addressing the limitations imposed by the small data set sizes typical for preclinical studies.

## Supplementary Material



## Notes

Supplemental figures illustrating model training dynamics, phantom validation results, and performance comparisons between clinical and preclinical transfer (PDF)
