# Supplementary material for: Cross-Modality Deep Learning Denoising for Low-Dose μSPECT: Transfer of PET-Trained U‑Net and Diffusion Models
Source: Chem Biomed Imaging. 2026 Mar 26;4(8):1727–39. doi: 10.1021/cbmi.5c00270 (PMC13508464; doi:10.1021/cbmi.5c00270)
Supplement: Supplementary file 1 [file im5c00270_si_001.pdf]

Supporting Information for

Cross-Modality Deep Learning Denoising for Low-Dose μSPECT: Transfer of PET-Trained U-Net and Diffusion Models

Elise Taragola[1*], Sara Neyt[1], Boxiao Yu[2], Maya Abi Akl[1], Boris Vervenne[1], Kuang Gong[2], Stefaan Vandenberghe[1], Christian Vanhove[1], and Florence M. Muller[1,3]

[1]Medical Image and Signal Processing, Department of Electronics and Information Systems, Faculty of Engineering and Architecture, Ghent University, Ghent, Belgium
[2]J. Crayton Pruitt Family Department of Biomedical Engineering, University of Florida, Gainesville, FL, USA
[3]Department of Radiology, Perelman School of Medicine, University of Pennsylvania, PA, USA

*Corresponding author's email: Elise.Taragola@UGent.be

Training and validation learning curves

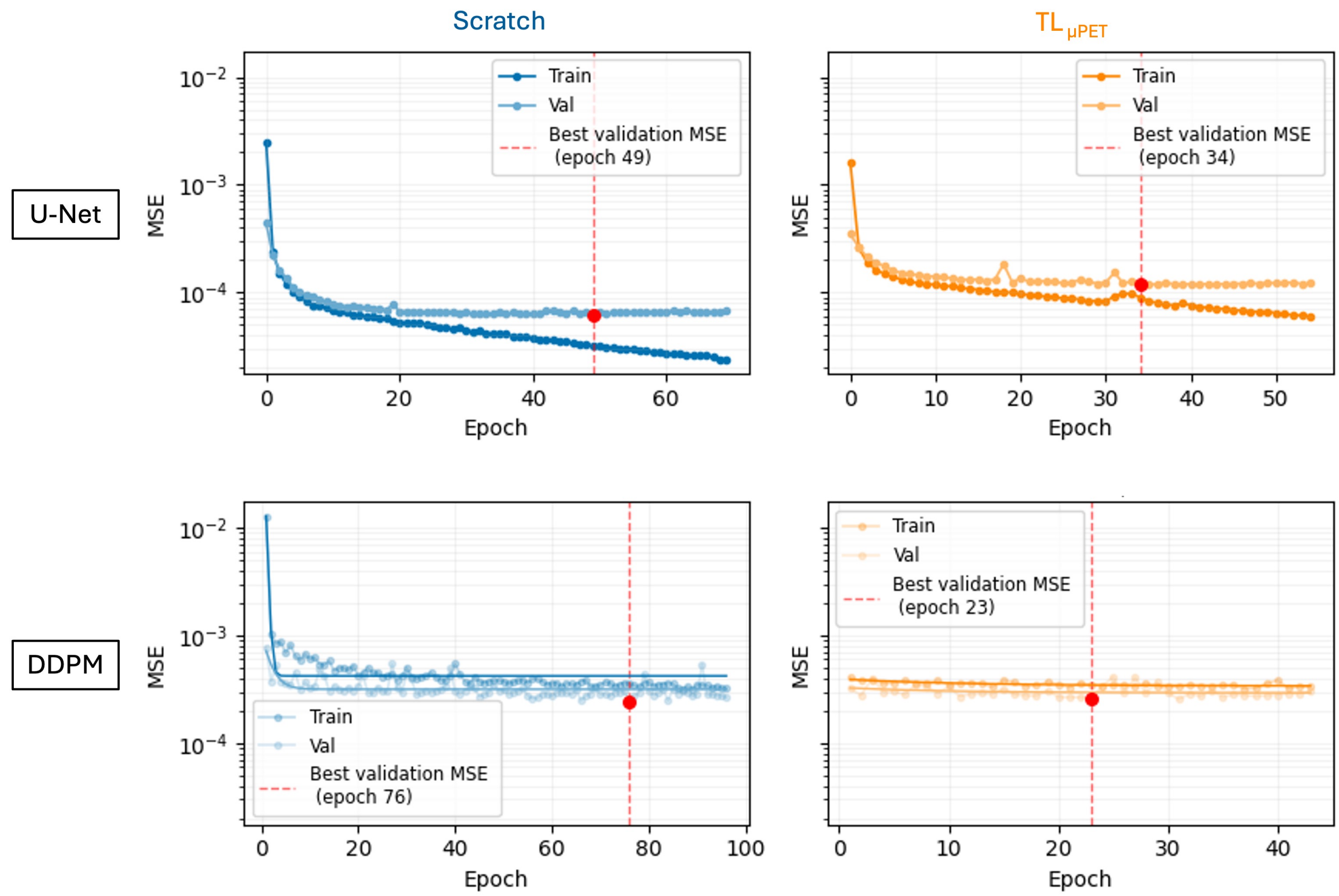

Figure S1: Representative training and validation learning curves (MSE per epoch) for the U-Net and DDPM models. Curves are shown for models trained from scratch (left column) and fine-tuned via transfer-learning ($TL_{\mu PET}$) on all μSPECT training data (right column). The red markers and dashed lines denote the epoch at which the lowest validation error was achieved, determining the model weights saved via early stopping. As illustrated in these curves, the initial validation loss for the transfer-learned models starts at substantially lower value compared to the models trained from scratch.

Phantom results

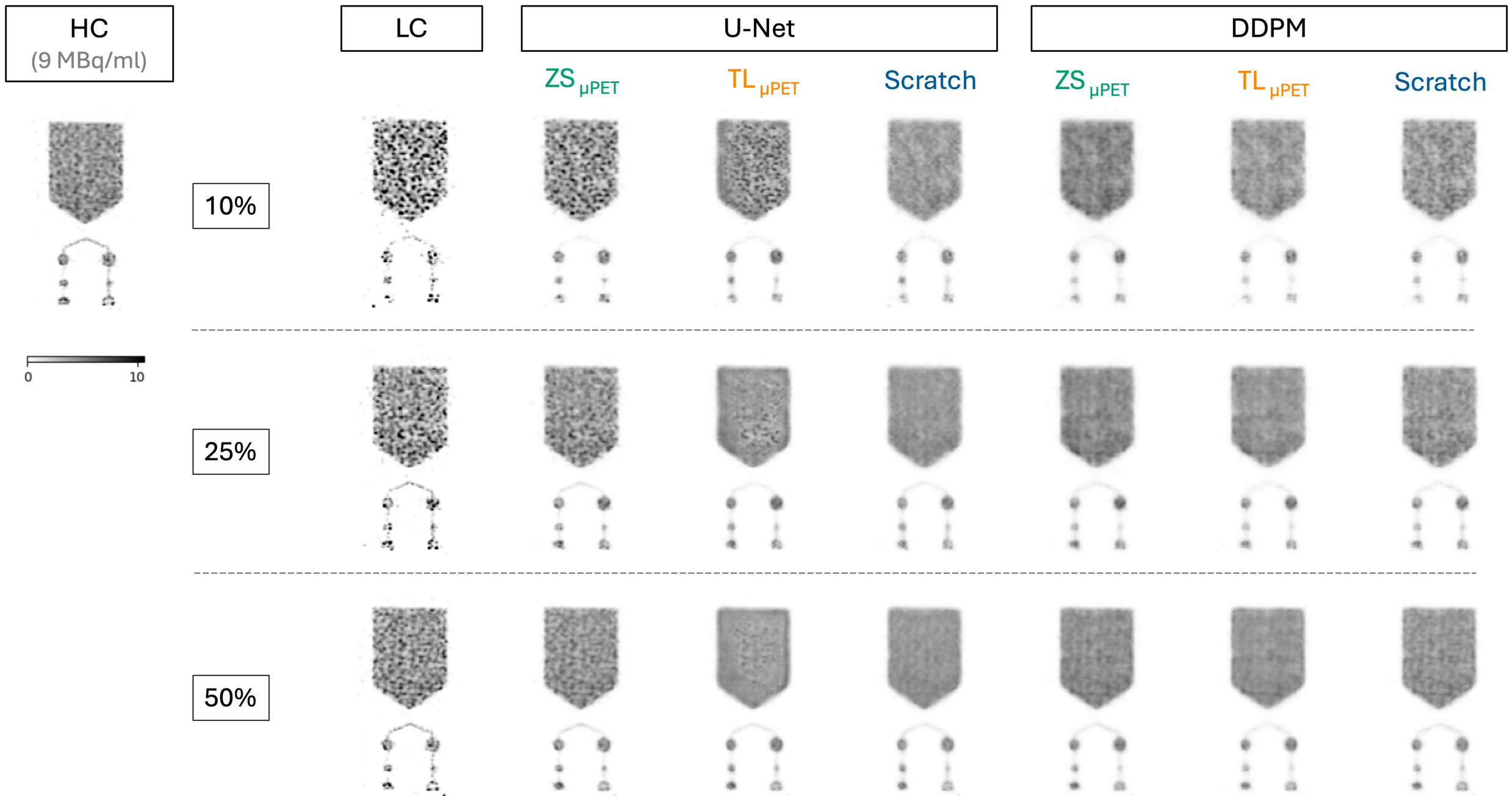

Figure S2: Cross-section of the Molecubes QC phantom. The high-count (HC) reference (injected activity: 9 MBq/ml) is shown together with the corresponding low-count (LC) inputs at 10%, 25%, and 50% and the zero-shot, transfer-learned, and scratch-trained U-Net and DDPM images.

Comparison of clinical and preclinical source domains

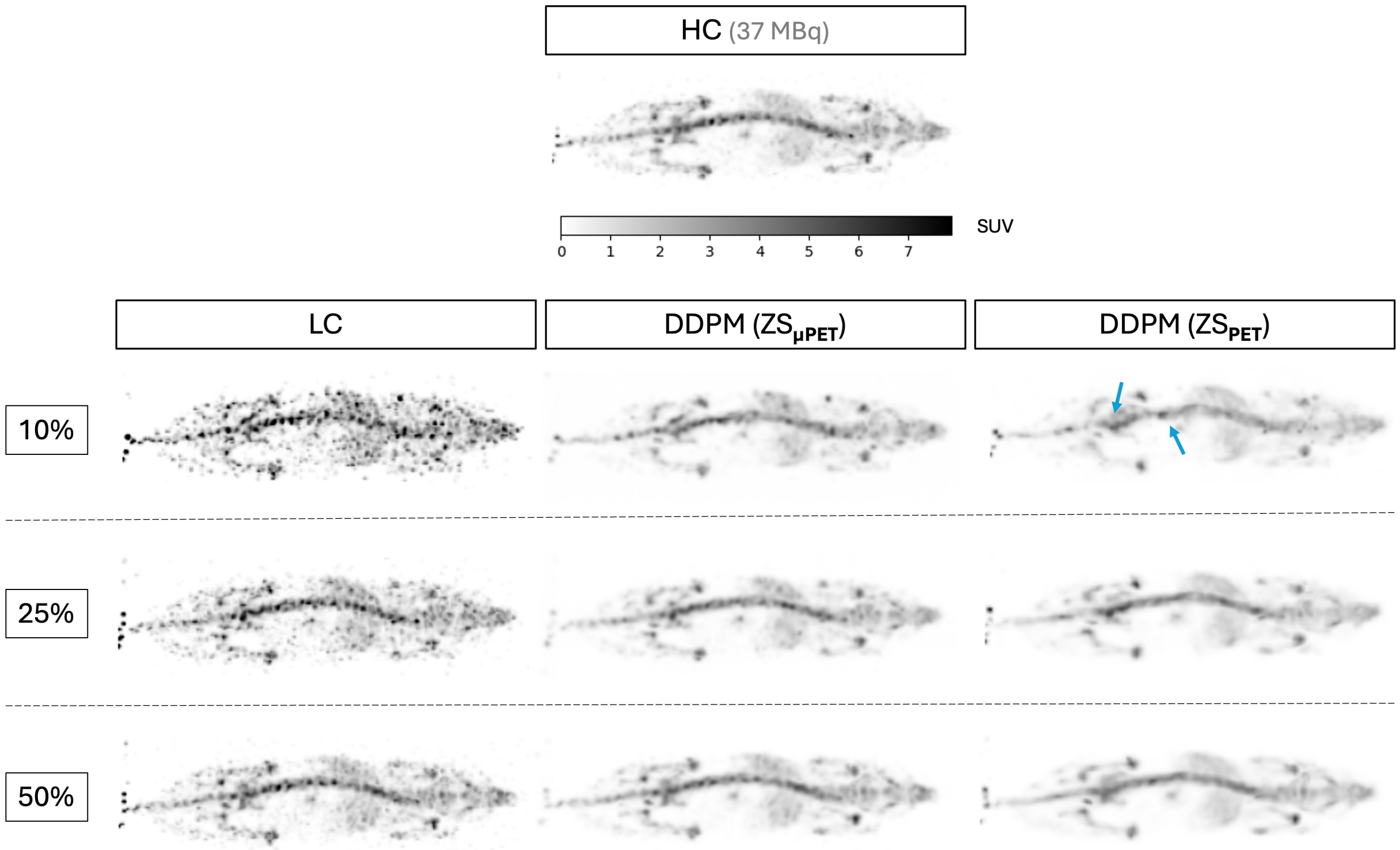

Figure S3: Maximum intensity projection images of a mouse ^{99m}Tc-HDP scan acquired three hours post-injection. The high-count (HC) reference (injected activity: 37 MBq) is shown together with the corresponding low-count (LC) inputs at 10%, 25%, and 50% and the zero-shot DDPMs pretrained on preclinical and clinical PET data. The blue arrows indicate distortions in the spine contour.

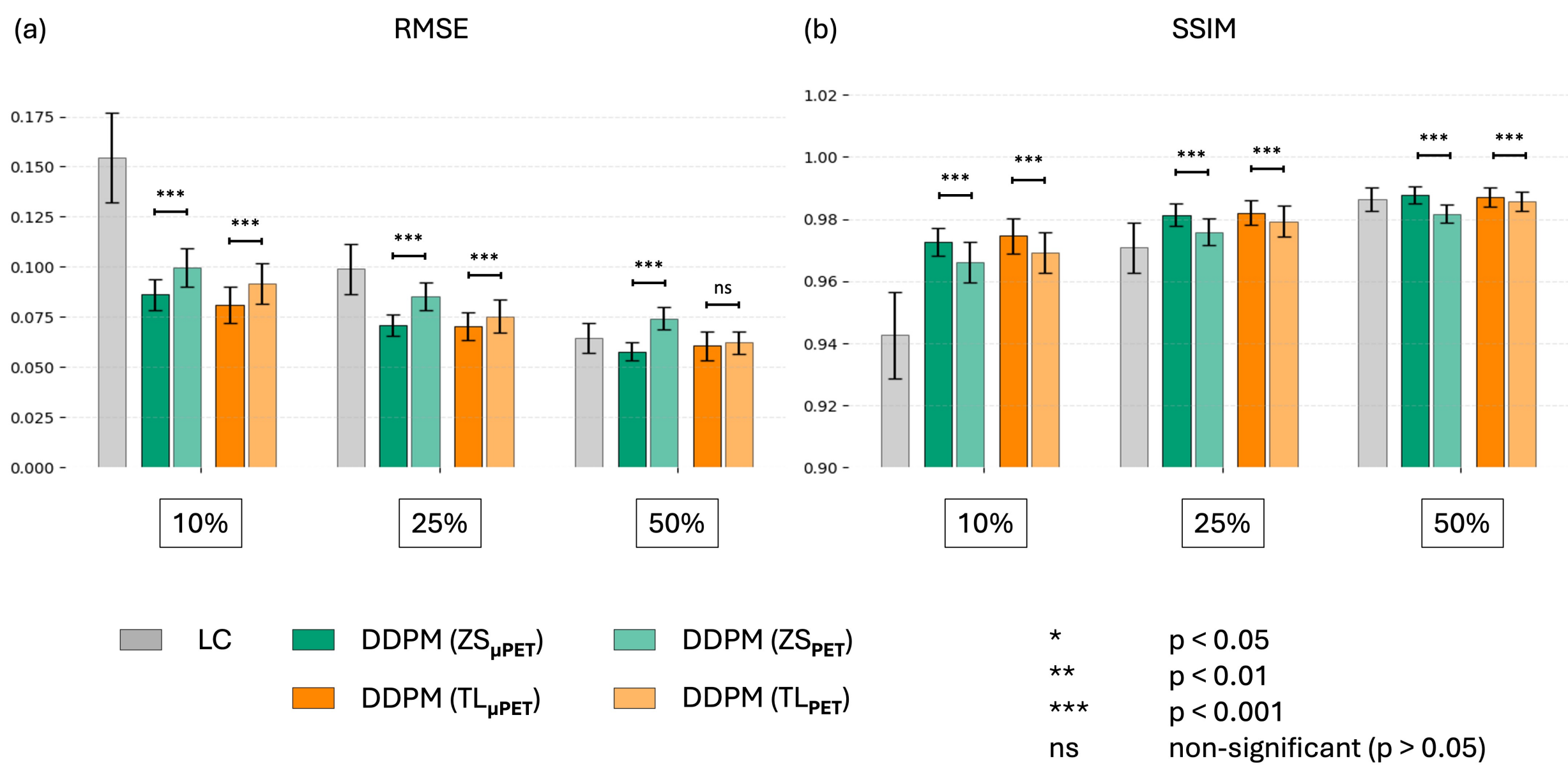

Figure S4: Quantitative comparison of preclinical and clinical source domains for zero-shot inference and transfer-learning. Volume-level (a) RMSE and (b) SSIM were evaluated for low-count (LC) inputs and DDPM outputs using two different pretraining domains: preclinical μPET (mouse) and clinical PET (human). Results are shown for 10%, 25%, and 50% count levels, computed across 11 test volumes. Each bar represents the mean metric value over subjects, with error bars indicating the standard deviation. Pairwise statistical comparisons between methods are reported for each count level.
